# Red blood cell distribution width as a novel marker for predicting bleeding after endoscopic resection for early gastric cancer

**DOI:** 10.1002/deo2.123

**Published:** 2022-05-13

**Authors:** Yumemi Takada, Shinji Yoshii, Tsukasa Yamakawa, Yuki Hayashi, Takakazu Miyake, Yoshihiro Yokoyama, Gota Sudo, Kei Mitsuhashi, Masanori Nojima, Hiro‐o Yamano, Hiroshi Nakase

**Affiliations:** ^1^ Department of Gastroenterology and Hepatology Sapporo Medical University School of Medicine Hokkaido Japan; ^2^ Department of Gastroenterology Hakodate Goryokaku Hospital Hokkaido Japan; ^3^ Department of Gastroenterology TOKEIDAI Memorial Hospital Hokkaido Japan; ^4^ Center for Translational Research The Institute of Medical Science Hospital The University of Tokyo Tokyo Japan

**Keywords:** endoscopic mucosal resection, endoscopic submucosal dissection, gastrointestinal hemorrhage, red cell distribution width, stomach neoplasm

## Abstract

**Objectives:**

Endoscopic resection (ER) is a minimally invasive treatment for early gastric cancer (EGC); however, there is a high occurrence of bleeding. This study aimed to clarify the significance of red blood cell distribution width (RDW) as a predictive risk factor for bleeding after ER for EGC.

**Methods:**

We conducted a retrospective study based on data for patients who underwent ER for EGC from 2019 to 2021. This study included 79 lesions in 54 patients who underwent ER for EGC. The primary outcome was the association between RDW before ER and bleeding within 28 days of treatment. Receiver operating characteristic (ROC) curves were constructed, wherein areas under the curve (AUCs) and 95% confidence intervals were calculated to compare the discriminatory power of RDW for predicting bleeding.

**Results:**

Endoscopic submucosal dissection was used as the resection method for 73 lesions, whereas endoscopic mucosal resection was used for six lesions. En bloc resection was performed in all cases. There were no cases of perforation; however, bleeding after ER occurred in five cases (9.3%). ROC curve analysis of bleeding after ER showed that the AUC was 0.843 with a good diagnostic performance. When the cut‐off value of RDW was set at 14.4%, sensitivity and specificity were 80% and 85.7%, respectively. There was a bleeding rate of 36.4% (4/11) at an RDW of ≥14.4%, which was significantly higher than that of 2.3% (1/43) at an RDW of <14.4%.

**Conclusion:**

RDW can be a predictor of bleeding risk after ER for EGC.

## INTRODUCTION

Endoscopic resection (ER), which includes techniques such as endoscopic submucosal dissection (ESD) and endoscopic mucosal resection, is the preferred treatment for early gastric cancer (EGC) since it is less invasive and more cost‐effective than traditional surgery.^1^, ^2^ Moreover, the risk of lymph node metastasis is low; however, bleeding after ER occurs in approximately 5% of cases.^3^ If bleeding occurs after ER, emergency hemostasis or blood transfusion is required. In some cases, hemorrhagic shock can be fatal, and highly invasive treatment, such as surgery and interventional radiotherapy, may be required. Therefore, if endoscopists could predict the risk of gastrointestinal (GI) bleeding related to ER, prophylactic hemostasis could be performed and carefully monitored for the early detection of bleeding in high‐risk patients.^4^ Recently, predictive models combining risk factors for GI bleeding and bleeding after endoscopic procedures have been reported by the Bleeding after ESD Trend from Japan (BEST‐J).^3^ The BEST‐J model stratifies bleeding risk into low, intermediate, high, and very high groups. The bleeding rates after ER were 2.8%, 6.1%, 11.4%, and 29.7%, respectively. These results indicate that the risk of bleeding is more than 10 times higher in very high‐risk patients than in low‐risk patients. However, 10 factors must be analyzed in this scoring system, and even in high‐risk patients, the bleeding risk is only approximately 10%. Therefore, a simple index or marker that can predict post‐ER bleeding would be useful for endoscopists.

Red blood cell distribution width (RDW) is a routine measurement of the red blood cell volume distribution in the complete blood cell count (CBC). Since this parameter is a component of CBC, its measurement is simple and inexpensive, and the results could be quickly obtained.^5^ It reflects the discrepancy in the size of the red blood cells, suggesting that RDW reflects the diagnosis and clinical outcome of GI diseases.^6^ If RDW can be a simple index for predicting post‐ER bleeding, it will have a significant clinical impact.^5^ However, no report has indicated its significance as a biomarker for predicting bleeding risk after ER in EGC.

This study aimed to clarify the significance of RDW as a biomarker for predicting bleeding risk after ER in EGC.

## METHODS

### Study design and patients

This study was conducted in accordance with the guidelines of the Declaration of Helsinki and was approved by the Institutional Review Board of Sapporo Medical University (No. 332–65). All data were retrospectively collected from the electronic medical records of patients who underwent ER for EGC between July 2019 and January 2021 at Sapporo Medical University Hospital. Routine blood tests – CBC that included hemoglobin, hematocrit, and RDW; standard biochemical tests; and coagulation tests – were performed in all patients with EGC within 1 week before ER. RDW is usually calculated by dividing the standard deviation of the mean corpuscular volume by the mean corpuscular volume and multiplying by 100 to yield a percentage value for RBC size heterogeneity. The following clinical data were obtained: patient age; sex; current medication history of aspirin, P2Y12 receptor antagonist, cilostazol, warfarin, direct oral anticoagulant, proton pump inhibitors/potassium‐competitive acid blockers, and steroids; comorbidities such as hypertension, cardiovascular diseases, liver diseases, diabetes mellitus, and chronic kidney disease with hemodialysis; endoscopic findings including lesion location, lesion diameter, presence of multiple lesions; and interruption of each type of antithrombotic agent. Moreover, the following data on the results of treatment were obtained: en bloc resection; complications such as perforation and bleeding after ER; and treatment for bleeding including hemostatic therapy, such as endoscopic coagulation, endoscopic clipping, transfusion, and surgery. Written informed consent for ER was obtained from all patients before ER. The need for informed consent of this study was waived via the opt‐out method on our hospital website.

### ER procedure

ESD was performed according to a previously reported standard procedure.^2^ Hemostatic forceps or clips were used during the procedure to actively stop bleeding and prophylactically coagulate the visible vessels of the ESD ulcer. The procedures were performed according to the guidelines of the Japanese Society of Japan Gastroenterological Endoscopy Society.^1^ A second‐look endoscopy was scheduled to be performed the day after ER. All patients received proton pump inhibitors and potassium‐competitive acid blockers during and after ER. For patients taking antithrombotic agents, the decision to continue or discontinue antithrombotic agents before ER and the timing of discontinuation, if any, was based on the Japanese guidelines.^7^

### Outcomes

The primary outcome was the association between the RDW value before endoscopic treatment and bleeding within 28 days of treatment. According to a previous report,^8^ bleeding after ER was defined as hemorrhage with clinical symptoms (hematemesis, melena, and hematochezia) or a decrease in hemoglobin of >2 g/dl since the patient's most recent laboratory test. Bleeding was confirmed by performing an emergency endoscopy from the time of the completion of ESD to 28 days after ER. Preventive hemostasis of visible vessels without evidence of bleeding during second‐look endoscopy was not regarded as bleeding after ER. The secondary outcome was a comparison of the predictive ability of RDW and BEST‐J scores for bleeding after ER. The Best‐J score consists of the following 10 factors: 4 points for Warfarin and direct oral anticoagulant (DOAC), 3 points for chronic kidney disease with hemodialysis, 2 points for P2Y12RA and aspirin, 1 point for cilostazol, tumor diameter >30 mm, lower‐third in tumor location and presence of multiple lesions, and ‐1 point for AT agent interruption. They were classified as low‐risk (0–1 point), intermediate‐risk (2 points), high‐risk (3–4 points), and very high‐risk (≥5 points).

### Statistical analysis

Categorical variables were summarized as n (%) and were compared using Fisher's exact tests. Univariate logistic regression analyses were used to test the associations of each variable with bleeding after ER. We constructed receiver operating characteristic (ROC) curves, wherein areas under the curves (AUCs) and 95% confidence intervals were calculated to compare the discriminatory power of the RDW level for predicting high‐risk patients. Furthermore, we compared the predictive ability of RDW and BEST‐J score for bleeding after ER. All data were processed and analyzed using JMP 16.1.0 (2020–2021 SAS Institute Inc., Cary, NC, USA) and EZR (Saitama Medical Center, Jichi Medical University, Saitama, Japan), which is a graphical user interface for R (The R Foundation for Statistical Computing, Vienna, Austria). Statistical significance was set at a *p*‐value <0.05.

## RESULTS

### Baseline clinical characteristics

This study included 79 lesions from 54 patients who underwent ER for EGC at our institute from July 2019 to January 2021. Of the 54 patients, there were 39 males and 15 females, with a median age of 74 years. 25 patients (46%) were over 75 years of age. Ten patients (18.5%) took one type of antiplatelet or anticoagulant medications, whereas 16 patients (29.6%) took two types of both medications. All patients taking antithrombotic medications were withdrawn according to the Japanese Society of Japan Gastroenterological Endoscopy Society guidelines. Twenty‐two patients (40.7%) took proton pump inhibitors and potassium‐competitive acid blockers for their underlying diseases, whereas five patients (9.3%) took steroids. Only one patient underwent hemodialysis (Table 1). The lesion was located in the lower third in 56.7% of the total cases. The median lesion diameter was 14.5 mm, with more than 30 mm in nine cases (16.7%). All lesions were EGC with T1a (87.1%) and differentiated adenocarcinoma (91.9%; Table 2).

**TABLE 1:** Patient characteristics

Patients	54
Age (years), mean (SD)	74 ± 7.51
Age categorized, no. (%)	
<75 years	25 (46)
≧75 years	29 (54)
Male, no. (%)	39 (72.2)
Active medication, no. (%)	
One type of AT	10 (18.5)
Two types of ATs	16 (29.6)
Aspirin	10 (18.5)
Clopidogrel	1 (1.9)
Cilostazol	1 (1.9)
Warfarin	2 (3.7)
DOAC	7 (13.0)
PPI × PCAB	22 (40.7)
Steroid	5 (9.3)
Comorbidities, no. (%)	
Hypertension	37 (68.5)
Cardiovascular diseases	12 (22.2)
Liver diseases	7 (18.5)
Diabetes	9 (16.7)
Hemodialysis	1 (1.9)

Age, male sex, active medication (single antiplatelet or anticoagulant, double antiplatelet or anticoagulant, aspirin, clopidogrel, cilostazol, warfarin), comorbidities (hypertension, cardiovascular diseases, liver diseases, diabetes, hemodialysis)

Abbreviations: AT, antithrombotic agent; DOAC, direct oral anticoagulant; PCAB, potassium‐competitive acid blockers, steroid; PPI, proton pump inhibitor.

**TABLE 2:** Lesion characteristics

Lesions, no.	79
Lesion location, no. (%)	
Upper‐third	11 (16.4)
Middle‐third	18 (26.9)
Lower‐third	38 (56.7)
Lesion diameter, mean (SD)	14.5 ± 13.7
Lesion diameter categorized, no. (%)	
<30 mm	45 (83.3)
≧30 mm	9 (16.7)
Multiple lesions, no. (%)	13 (24.1)
Macroscopic type, no. (%)	
0–I	4 (5.1)
0–IIa	27 (34.6)
0–IIb	5 (6.4)
0–IIc	24 (30.8)
0–IIa + IIc	12 (15.4)
Invasion depth, no. (%)	
T1a	54 (87.1)
T1b	8 (12.9)
Ulceration, no. (%)	9 (16.7)
Vascular invasion, no. (%)	1 (1.8)
Tumor differentiation, no. (%)	
Differentiated (well, mod, pap)	57 (91.9)
Undifferentiated (sig, por, muc)	5 (8.1)

Abbreviations: mod, moderately‐differentiated tubular adenocarcinoma; muc, mucinous adenocarcinoma; pap, papillary adenocarcinoma; por, poorly‐differentiated tubular adenocarcinoma; SD, standard deviation; sig, signet ring cell carcinoma; well, well‐differentiated tubular adenocarcinoma.

ESD was used as the resection method in 73 lesions, whereas endoscopic mucosal resection was used in six lesions. En bloc resection was performed in all patients. The procedure time of more than 120 min was observed in eight cases (14.8%). Second‐look endoscopy was performed in 92.5% of the cases. There were no cases of perforation; however, bleeding after ER occurred in five cases (9.3%; Table 3). Table S1 shows the details of the five bleeding cases. Blood tests results showed that the median RDW was 13.1% (12.7%–14.0%). There were no abnormalities in any other findings (Table 4).

**TABLE 3:** Endoscopic procedure and outcomes

Resection method, no. (%)	
ESD	73 (92.4)
EMR	6 (7.6)
En bloc resection, no. (%)	79 (100)
Procedure time (min), no. (%)	
≧120 min	8 (14.8)
Second‐look endoscopy	50 (92.5)
Adverse events	
Perforation, no. (%)	0 (0)
Bleeding, no. (%)	5 (9.3)

Abbreviations: EMR, endoscopic mucosal resection; ESD, endoscopic submucosal dissection.

**TABLE 4:** Laboratory data

	Median (IQR)
WBC (×10^3^/μl)	5.8 (4.7–6.8)
RBC (×10^6^/μl)	4.4 (4.0–4.8)
Hb (g/dl)	13.7 (12.7–14.6)
MCV (fl)	93.5 (90.2–96.5)
RDW (%)	13.1 (12.7–14.0)
Plt (×10^3^/μl)	214 (177–258)
Alb (g/dl)	4.2 (4.0–4.4)
BUN (mg/dl)	18 (13–22)
eGFR (ml/min)	63.1 (56.3–75.5)
PT‐INR	0.97 (0.92–0.99)

Alb, albumin; BUN, blood urea nitrogen; eGFR, estimated glomerular filtration rate; Hb, hemoglobin; IQR, interquartile range; MCV, mean corpuscular volume; Plt, platelet; PT‐INR, prothrombin time‐international normalized ratio; RBC, red blood cell; RDW, red blood cell distribution width; WBC, white blood cell.

### Relationship between the RDW values and bleeding after ER

**FIGURE 1:**
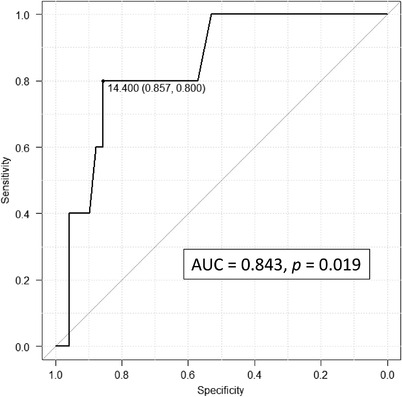
Receiver operating characteristic curve analysis for red blood cell distribution width. AUC, area under the curve

ROC curve analysis of bleeding after ER showed that the AUC was 0.843 (95% confidence interval [CI] 0.680–1, *p* = 0.019) with high diagnostic performance. Moreover, the optimal cut‐off value of RDW was investigated. When the cut‐off value of RDW was set at 14.4%, sensitivity and specificity were 80.0% and 85.7%, respectively (Figure 1).

The bleeding rate was 36.4% (4/11) at an RDW of ≥14.4%, which was significantly higher than that of 2.3% (1/43) at an RDW of <14.4% (*p* = 0.005).

Table S2 shows the univariate analysis of predictive factors for bleeding after ER for EGC. RDW and two antithrombotic agents, including DOAC and aspirin, were significantly associated with bleeding. The highest Odds ratio was 21.83 (95% CI 1.84–1198.1, *p* = 0.005) for RDW, followed by 16.88 (95% CI 2.15–132.51, *p* = 0.013) for DOAC and 9.0 (95% CI 1.27–63.89, *p* = 0.039) for aspirin.

### Relationship between the RDW values and BEST‐J score

**FIGURE 2:**
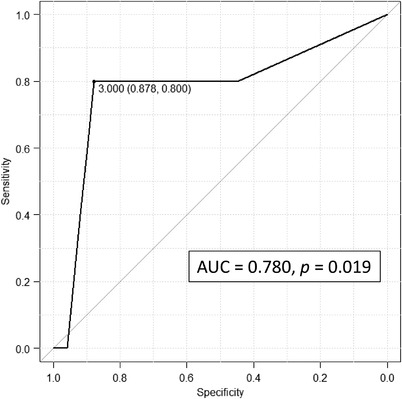
Receiver operating characteristic curve analysis for Bleeding after Endoscopic Submucosal dissection Trend from Japan score. AUC, area under the curve

**FIGURE 3:**
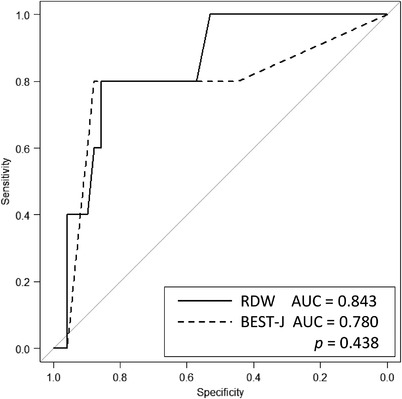
Receiver operating characteristic curve comparison between red blood cell distribution width (RDW) and Bleeding after Endoscopic Submucosal Dissection Trend from Japan score. AUC, area under the curve

According to BEST‐J, the low‐risk group consisted of 38 cases (70.4%), intermediate six cases (11.1%), high‐risk four cases (7.4%), and very‐high risk six cases (11.1%). ROC curve analysis was performed for bleeding after ER of BEST‐J. The AUC was 0.780 (95% CI 0.5–1.0, *p* = 0.017), with high diagnostic performance (Figure 2). ROC analysis compared the AUC value of RDW and BEST‐J score for bleeding after ER, showing no significant difference from RDW (*p* = 0.438; Figure 3).

## DISCUSSION

This study examined the role of RDW as a predictor of GI bleeding risk after ER for patients with EGC. We found that there was a bleeding rate of 36.4% (4/11) in patients with an RDW of ≥14.4%, which was significantly higher than 2.3% (1/43) in patients with an RDW of <14.4%. Moreover, the ROC curve for RDW (0.843) showed a statistically significant power, with a sensitivity and specificity of 80.0% and 85.7%, respectively. Our findings suggest that the RDW can be a simple predictive parameter that can predict the risk of bleeding after ER for EGC.

ER, including ESD and endoscopic mucosal resection, is a minimally invasive treatment for EGC; however, there is a high occurrence of GI bleeding.^3^ Risk stratification of bleeding after ER for EGC is challenging. The BEST‐J score is a predictive model for bleeding after ESD in a nationwide multicenter study. However, even if we apply the BEST‐J score, which is a well‐established method for predicting bleeding risk, the bleeding rate is only 10% in the high‐risk group. In this regard, identifying a simple index for predicting post‐ER bleeding is clinically important.

RDW is a measurable parameter that represents the degree of heterogeneity of red blood cells. Since this parameter is a component of CBC, its measurement is simple and inexpensive, and the results can be quickly obtained.^5^ Many reports have demonstrated that a high RDW may be closely associated with increased morbidity and mortality in various types of malignancy and diseases, such as diabetes mellitus, cardiovascular, renal, thromboembolic, respiratory, liver, and inflammatory diseases.^9^, ^10^, ^11^, ^12^, ^13^, ^14^, ^15^, ^16^, ^17^, ^18^ Furthermore, it has been reported that a drastic increase in RDW is a strong predictor of patient fatality.^19^ Other reports have shown that RDW has an independently linear relationship with recurrent and massive bleeding in severe conditions, such as post‐percutaneous coronary intervention, intracranial hematoma, and multiple trauma patients.^18^, ^19^, ^20^ A recent study showed that a high RDW (≥14.5%) was strongly associated with a high risk of upper GI bleeding.^5^

This study suggests that RDW is a predictor of risk for GI bleeding in patients who underwent ER for EGC. There may be several reasons why high RDW contributes to GI bleeding with ER. Erythropoietin is a significant determinant of RDW, and increased RDW is affected by abnormal erythropoietin production and hypofunction of the erythropoietin response.^20^, ^21^ The possible mechanism associated with elevated RDW, a pathological process involving inflammation and thrombotic effects in blood vessels, maybe inhibit erythrocyte maturation via inflammatory cytokines such as interleukin (IL)‐1, IL‐6, and tumor necrosis factor α.^22^, ^23^ These inflammatory factors can disrupt iron utilization and reduce the responsiveness of bone marrow in response to erythropoietin. They can also inhibit anti‐apoptotic effects and stimulate the maturation of cells. In turn, they increase the number of immature cells released into the peripheral circulation and increase the heterogeneity of red blood cells. These mechanisms support the hypothesis that a high RDW may be associated with a tendency for upper GI bleeding.

Our study with a few cases suggested that the RDW has a possible diagnostic performance comparable to the BEST‐J score. BEST‐J mainly reflects antithrombotic agents and lesion characteristics. Conversely, RDW reflects patient characteristics associated with inflammatory factors. BEST‐J and RDW may evaluate bleeding risk from different aspects. Therefore, we believe that the combination of BEST‐J and RDW can help detect more high‐risk patients in the future.

This pilot study with a small number of patients was conducted at a single institution. Therefore, this study had several limitations. First, we could not perform a multivariate analysis to evaluate whether RDW is the most useful factor, including other factors. Second, appropriate cut‐off values for RDW could not be established due to different normal values for different devices and genders. To address these issues, we need to validate our method using another dataset with a larger number of cases.

In conclusion, a high RDW may predict bleeding after ER for EGC. Moreover, prophylactic hemostasis and careful monitoring are necessary for patients with high RDW.

## CONFLICT OF INTEREST

The authors declare no conflict of interest.

## FUNDING INFORMATION

None.

## Supporting information


**Table S1**. Details of the characteristics of five cases of bleeding after endoscopic resection.
**Table S2**. Univariate analysis of predictive factors for bleeding after endoscopic resection for early gastric cancer.
