# Correction to “Red blood cell distribution width as a novel marker for predicting bleeding after endoscopic resection for early gastric cancer”

**DOI:** 10.1002/deo2.280

**Published:** 2023-08-07

Takada Yumemi, Shinji Yoshii. DEN Open. 2022 May 13;3(1)

In author name, “Takada Yumemi” was incorrect. This should have read: “Yumemi Takada”.

We apologize for this error.

