# Supplementary material for: Red blood cell distribution width as a novel marker for predicting bleeding after endoscopic resection for early gastric cancer
Source: DEN Open. 2022 May 13;3(1):e123. doi: 10.1002/deo2.123 (PMC9549875; doi:10.1002/deo2.123)
Supplement: Supplementary file 1 — Table S1. Details of the characteristics of five cases of bleeding after endoscopic resection. Table S2. Univariate analysis of predictive factors for bleeding after endoscopic resection for early gastric cancer. [file DEO2-3-e123-s001.docx]

**Supplementary Table 1.** Details of the characteristics of five cases of bleeding after ER.

|  | Case 1 | Case 2 | Case 3 | Case 4 | Case 5 |  |
| --- | --- | --- | --- | --- | --- | --- |
| Time interval between ESD and bleeding | Day 1 | Day 11 | Day 8 | Day 1,11 | Day 8 |  |
| RDW | 17.3 | 14.4 | 17.9 | 14.6 | 14.2 |  |
| BEST-J | 3 | 6 | 4 | 5 | 3 |  |
| (risk) | (high) | (very-high) | (high) | (very-high) | (high) |  |
| Antithrombotic agents | aspirin | aspirin | aspirin | DOAC | None |  |
|  |  | DOAC | DOAC |  |  |  |
| Comorbidities | Vasculitis | Ischemic heart disease | Atrial Fibrillation | Angina pectoris | Autoimmune hepatitis |  |
|  |  |  |  |  |  |  |
|  |  |  |  |  |  |  |
| Other risk | Lesion diameter 65mm | Lesion location Lower-third | None | Multiple lesion Lesion diameter 30mm | Multiple lesion　 Lesion diameter 34mm Lesion location Lower-third |  |
|  |  |  |  |  |  |  |
|  |  |  |  |  |  |  |

� BEST-J; Bleeding after ESD Trend from Japan, RDW; red blood cell distribution width; DOAC, direct oral anticoagulant

**Supplementary Table 2.** Univariate analysis of predictive factors for bleeding after ER for EGC

| Item | odds ratio | 95% CI | P-value |
| --- | --- | --- | --- |
| age (over 75) | 0.75 | 0.12-4.91 | 0.767 |
| sex (M/F) | n/a | n/a | n/a |
| antithrombotic agents |  |  |  |
| **aspirin** | **9.0** | **1.27-63.89** | **0.039** |
| Clopidogrel | n/a | n/a | n/a |
| Cilostazol | n/a | n/a | n/a |
| Warfarin | n/a | n/a | n/a |
| **DOAC** | **16.88** | **2.15-132.51** | **0.013** |
| Cardiovascular diseases | 6.67 | 0.97-45.92 | 0.0668 |
| Hemodialysis | n/a | n/a | n/a |
| Multiple lesions | 0.77 | 0.078-7.58 | 0.823 |
| Tumor size>30mm | 0.975 | 0.098-9.71 | 0.983 |
| Tumor location lower third | n/a | n/a | n/a |
| Tumor differentiation undifferentiated | n/a | n/a | n/a |
| Invasion depth, T1b | n/a | n/a | 0.603 |
| Ulceration | 0.78 | 0.077-7.93 | 0.810 |
| Procedure time ≧120 min | n/a | n/a | n/a |
| Resection type, piecemeal | n/a | n/a | 0.907 |
| 2nd look endoscopy | n/a | n/a | n/a |
| **RDW** | **21.83** | **1.84-1198.1** | **0.005** |

� RDW; red blood cell distribution width, DOAC, direct oral anticoagulant, n/a; not assessed
